# The perspectives of patients and their caregivers on self-management interventions for chronic conditions: a protocol for a mixed-methods overview

**DOI:** 10.12688/f1000research.22125.1

**Published:** 2020-02-18

**Authors:** Ena Niño de Guzmán, Laura Martínez García, Ana I. González, Monique Heijmans, Jorge Huaringa, Kaisa Immonen, Lyudmil Ninov, Carola Orrego-Villagrán, Javier Pérez-Bracchiglione, Karla Salas-Gama, Andrés Viteri-García, Pablo Alonso-Coello

**Affiliations:** 1 Iberoamerican Cochrane Centre, Sant Pau Biomedical Research Institute (IIB-Sant Pau), Barcelona, 08025, Spain; 2 Universitat Autònoma de Barcelona, Barcelona, Spain; 3 CIBER de Epidemiología y Salud Pública (CIBERESP), Madrid, Spain; 4 Avedis Donabedian Research Institute (FAD), Barcelona, 08037, Spain; 5 Red de Investigación en Servicios de Salud en Enfermedades Crónicas (REDISSEC), Madrid, Spain; 6 Netherlands institute for health services research (Nivel), Utrecht, 3513, The Netherlands; 7 Hospital Arzobispo Loayza, Lima, Lima 15082, Peru; 8 European Patients’ Forum, Brussels, 1040, Belgium; 9 Interdisciplinary Centre for Health Studies (CIESAL), Universidad de Valparaíso, Valparaíso, 2520000, Chile; 10 Hospital Santa Creu i Sant Pau, Barcelona, 08025, Spain; 11 Universidad UTE, Quito, 170527, Ecuador; 12 Cochrane Ecuador, Centro de Investigación en Salud Pública y Epidemiología Clínica (CISPEC), Quito, 170527, Ecuador

**Keywords:** Systematic Review, Self-Management, Chronic Diseases, Mixed-Methods Research, Patient Preferences, Outcomes

## Abstract

**Introduction**: Self-management (SM) interventions are complex interventions and one of the main components of high-quality chronic disease care for which the incorporation of the perspectives of patients and their informal caregivers is crucial. We aim to identify, appraise and synthesise the evidence exploring patients’ and caregivers’ perspectives on SM interventions. More precisely, we aim to 1) describe how they value the importance of outcomes of SM interventions, and 2) identify the factors that might impact on acceptability and feasibility of SM interventions based on their preferences and experiences.

**Methods and analysis**: We will conduct four mixed-methods overviews as part of COMPAR-EU, a European Union (EU) funded project aimed to identify the most effective and cost-effective SM interventions for chronic obstructive pulmonary disease (COPD), heart failure (HF), obesity, and type 2 diabetes mellitus (T2DM). We will search in MEDLINE, CINAHL, and PsycINFO for systematic reviews of studies addressing patients’ preferences on outcomes, or their experiences with SM alongside their disease trajectory or with SM interventions, published in English. Selection of studies and data extraction will be conducted in pairs. We will assess the overlap of studies and methodological quality. We will follow a three-step synthesis process: 1) narrative synthesis for quantitative evidence, 2) thematic synthesis for qualitative evidence, and 3) integration of findings in the interpretation phase. Additionally, we will consult on the relevance of findings with patients and their caregivers.

**Systematic review registration**: PROSPERO CRD42019117867

## Introduction

The global burden of chronic conditions constitutes a significant public health challenge that undermines social and economic development, accounting for almost 86% of deaths and 77% of the disease burden in the WHO European Region. The increasing healthcare demands of chronic conditions lead to the development of integrated healthcare models based on patient-centred care ^1^.

Self-management (SM) interventions are critical components of high-quality patient-centred care in chronic conditions ^1– 3^. SM can be defined as what individuals, families, and communities do to promote, maintain, or restore health and to cope with the disease or disability with or without the support of health professionals. It includes, but is not limited to, self-prevention, self-diagnosis, self-medication, and daily management of the disease and its disabilities ^4^. Ideally, SM interventions support patients taking their individual needs and their specific context into account ^5^. SM interventions are complex and multifaceted and may contain multiple topics, formats, and components (e.g. participants, family, facilitators, or technology) that interact with each other. Different outcomes can be defined to measure their impact based on the specific scope (e.g. behavioural, psychosocial, physiological, or utilisation outcomes) of the intervention ^5, 6^.

An increasing body of evidence suggests that SM interventions have beneficial effects for patients with chronic conditions in terms of knowledge, the performance of SM behaviours, self-efficacy, health status ^7^ and quality of life ^8^. However, what might be beneficial for some patients might be less beneficial for others; for that reason, it is necessary to identify which elements of SM interventions are effective and under what circumstances. Especially, it is important to consider the perspectives of patients and their caregivers to get insight into their values and preferences on specific healthcare alternatives ^9– 11^.

Preference is conceived as the result of cognition, experience and reflection. Moreover, a specific preference is the consequence of what a person values ^12^. In healthcare, patients’ preferences can be defined as the qualitative or quantitative assessments of the relative desirability or acceptability to patients of specified alternatives or choices among outcomes or other attributes that differ among alternative health interventions ^13^.

The GRADE Evidence to Decision (EtD) framework is a decision-making tool that integrates the perspective of patients under the term *values,* defined as the relative importance of outcomes or health states of interest ^14^. The patient’s preference for or against intervention is an implicit result of the relative importance of the health outcomes an individual connects to the intervention ^14^. The patient’s preference is also implicitly related to other aspects such as attitudes, expectations and beliefs. These aspects often fall within two other EtD criteria: *acceptability*, whether interventions are acceptable for patients; and *feasibility*, whether interventions are feasible to implement from the patient’s point of view ^13– 15^. However, the methods to identify, collect and integrate this body of evidence in healthcare decision-making is still an evolving research area ^10^ which requires more practical guidance ^16, 17^.

One method to find out the perspectives of patients and their caregivers is the development of a systematic review (SR) of studies exploring how they value the importance of outcomes or the features of the interventions that might affect their benefit-risk perceptions. This information can be obtained from quantitative and qualitative evidence reported as utility or non-utility measures ( Table 1) ^9, 14, 18^. Utility measures reflect how a respondent values or feels about a state of health ^19^. Non-utility measures inform about patients’ (or their informal caregivers’) preferences and experiences with the intervention of interest derived from quantitative, qualitative or mixed methods studies ^14^.

**Table 1.** Methods to ascertain values and preferences.

Measures	Source /type of data	Method
**Health utilities**	**Direct**	**Matching** - Allocation game - Standard gamble - Time trade-off - Visual analogue scale - Willingness to pay **Conjoint analysis** - Best-worst choice experiment - Binary choice experiments - Conjoint analysis - Full ranking exercise - Multinomial choice experiment - Probability trade-off
	**Indirect**	**Multi-attribute utility instruments** - EuroQoL-5D - Health Utilities Index 2S - Short-form-6D - PROMIS-29 **Quality of life tools** - EQ5D utility estimates from St. George Respiratory Questionnaire - Others
**Non-utilities (preferences** **and experiences)**	**Quantitative**	- Adaptive questioning - Direct choice - Ranking - Rating (e.g. numerical rating scales) - Survey
	**Qualitative**	- Focus groups - Interviews

Adapted from Zhang *et al.* ^14^

Since 2010, there has been an increasing number of qualitative evidence synthesis (QES) published exploring patients’ and their caregivers' experiences and perceptions regarding living with a chronic condition, some of these explicitly addressing the process of self-care ^20^. There are also some SRs of studies exploring patients’ preferences quantitatively for different health states ^20^.

However, summarising this body of evidence in a complete and comprehensible way for stakeholders might be challenging. In this context, the development of overviews might represent the most suitable option to summarise this broad and complex phenomenon. Previously published overviews addressing this research area were very diverse in the length and depth of scope ^21– 25^. However, some focused more on SM interventions and included only qualitative evidence for some specific conditions, such as stroke ^24^, rheumatic diseases, cancer and fibromyalgia ^21^. Others explored the experience of living with a chronic condition using a quantitative ^22^ or qualitative approaches ^23^. Moreover, some were focused on some specific symptoms (e.g. chronic non-malignant pain ^25^). Even though previous research had enhanced knowledge on the perspectives of patients and their caregivers on chronic conditions and SM, we consider it is required to move towards a more comprehensive approach, applying a mixed-methods approach ^26^ including quantitative and qualitative sources of evidence, through developing overviews of systematic reviews.

### Objectives

This study protocol is part of a large multi-method and multi-step project: “Comparing the cost-effectiveness of self-management interventions in four high priority chronic diseases in Europe: COMPAR-EU”. This is a European Union-funded project aiming to identify the most effective and cost-effective SM interventions for patients living with chronic obstructive pulmonary disease (COPD), heart failure (HF), obesity or type 2 diabetes mellitus (T2DM).

Our main objective is to identify, appraise, and analyse the perspectives of patients and their caregivers on outcomes of SM interventions for the selected chronic conditions. To provide a more detailed assessment, we will conduct four overviews (one per chronic condition). Our specific objectives are 1) to explore how patients with one of the four chronic conditions, and their caregivers, value the importance of different outcomes of SM interventions, and 2) to identify the factors that might impact the acceptability and feasibility of SM interventions based on their preferences and experiences with SM through the disease trajectory and when taking part in SM interventions.

## Methods and analysis

We will conduct four mixed-methods overviews of SRs according to the most updated methodological guidance from the Cochrane Collaboration ^27– 29^, the Joanna Briggs Institute (JBI) ^30^ and key methodological references ^31– 34^. We adhere to the applicable items of the Preferred Reporting Items for Systematic reviews and Meta-Analyses Protocols (PRISMA-P) statement ^35^. We registered this protocol in the PROSPERO registry: CRD42019117867. Any amendment to the protocol and the supporting rationale will be reported.

### Eligibility criteria

***Type of reviews.*** We will consider for inclusion: 1) quantitative SRs 2) qualitative evidence syntheses QES; and 3) mixed methods research synthesis (MMRS). To be eligible for inclusion, the SRs should report as a minimum: 1) the search strategy for at least one database; 2) the list of included studies; and 3) the methods applied for synthesis ^36^.

***Context/setting.*** We will include SRs of studies without geographical restriction and will include all settings, except those confined to inpatient care.

***Population/perspectives.*** We will limit inclusion to SRs of studies conducted in adult patients (18 or older) with one or more of the chronic conditions of interest (COPD, HF, obesity and T2DM), without restricting by disease severity. We will consider SRs assessing multiple chronic conditions if data for one or more of the chronic conditions we are interested in are reported separately.

We will include SRs exploring the perspectives of patients or their informal caregivers. We will include SRs assessing both perspectives if these are reported separately. We will exclude SRs addressing only healthcare providers’ or healthy population perspectives.

***The phenomenon of interest/intervention.*** We will include SRs aimed at exploring the perspectives of patients and their caregivers on the importance of outcomes of SM interventions. Their perspectives are based on their values, preferences and experiences. As described by Zhang *et al.* ^14^, preferences for or against intervention are implicitly related to the relative importance people place (or value) on the expected or definite health outcomes connected to a specific intervention. We define SM intervention as supportive interventions systematically provided by healthcare staff, peers or laypersons to increase patients’ skills and confidence in their ability to manage a chronic condition. SM interventions aim to prepare patients (and, where appropriate, informal caregivers ^37^) to actively participate in the management of their disease ^37, 38^.

We will apply the COMPAR-EU taxonomy for the domain of outcomes of SM interventions, with 35 elements categorised in seven subdomains ^39^: 1) empowerment, 2) level of fulfilment of the expected SM behaviours, 3) clinical outcomes (progression of disease, complications, adverse events, and mortality), 4) quality of life of patients and caregivers, 5) perceptions and satisfaction with care, 6) health care use, and 7) cost. We will not exclude SRs addressing other outcomes not listed in the COMPAR-EU taxonomy. Instead, these will be incorporated as a new category if applicable.

***Comparison.*** While not addressed as an objective in these overviews, if reported, we will consider usual care as the comparison.

***Outcomes.*** We will include SRs evaluating studies reporting any of the following outcomes:

- Utility measures for outcomes of SM interventions; defined as how patients or their caregivers value the importance of different outcomes. Utilities are measures of the value ascribed to states of health, most often scaled from “0”, representing death, to “1”, representing perfect health; they reflect the health-related quality of life of the individual at a particular point in time ^40^. Methods to obtain utilities can be: a) direct methods such as matching methods (i.e. standard gamble, time trade-off, or rating scales), or conjoint analysis (i.e. discrete choice experiments, contingent valuation and willingness to pay, probability trade-off, paired comparison); or b) indirect methods with multi-attribute instruments like the EuroQual-5-dimension (EQ-5D), the Short Form-6-Dimension (SF-6D), Health Utilities Index (HUI-3) ^14^, the Patient-Reported Outcomes Measurement Information System (PROMIS-29) ^41^, among other quality of life tools which transform results across several domains into utilities ( Table 1).
- Non-utility measures for outcomes of SM interventions, which are the result of the assessment of preferences and experiences of patients and their caregivers with SM in the disease trajectory or with SM interventions through quantitative (e.g. surveys), qualitative (e.g. interviews, focus groups), or mixed methods studies ^14^ ( Table 1).

### Information sources and search strategy

We will design and execute a literature search strategy in MEDLINE (accessed through PubMed), the Cumulative Index of Nursing and Allied Health Literature (CINAHL), and PsycINFO. The leading search concepts will be: 1) the perspective of patients, for which we will include a sensitive content search strategy previously published ^42^; and 2) specific terms for each chronic condition. We will apply methodological filters limiting, to SRs, the retrieval of references using the filters available in each database, and to qualitative research, synthesis using non-controlled terms. We will consider English-language studies for inclusion without restriction on publication date. We will hand-search the reference list of overviews identified through our search strategy and will conduct a forward citation search of the included studies in Scopus ^43^. We will establish regular search update alerts. Studies identified through these alerts and meeting the eligibility criteria will be incorporated until starting the stakeholders’ consultation.

### Selection of studies

We will manage references with Endnote X9. Pairs of authors will conduct the screening of references and selection of studies. After initial calibration with 10% of retrieved references, one author will independently select references to be included, and a second author will check the final list of included and excluded studies with reasons. Disagreements will be solved by discussion or with the help of a third author.

### Data collection

**Figure 1.**
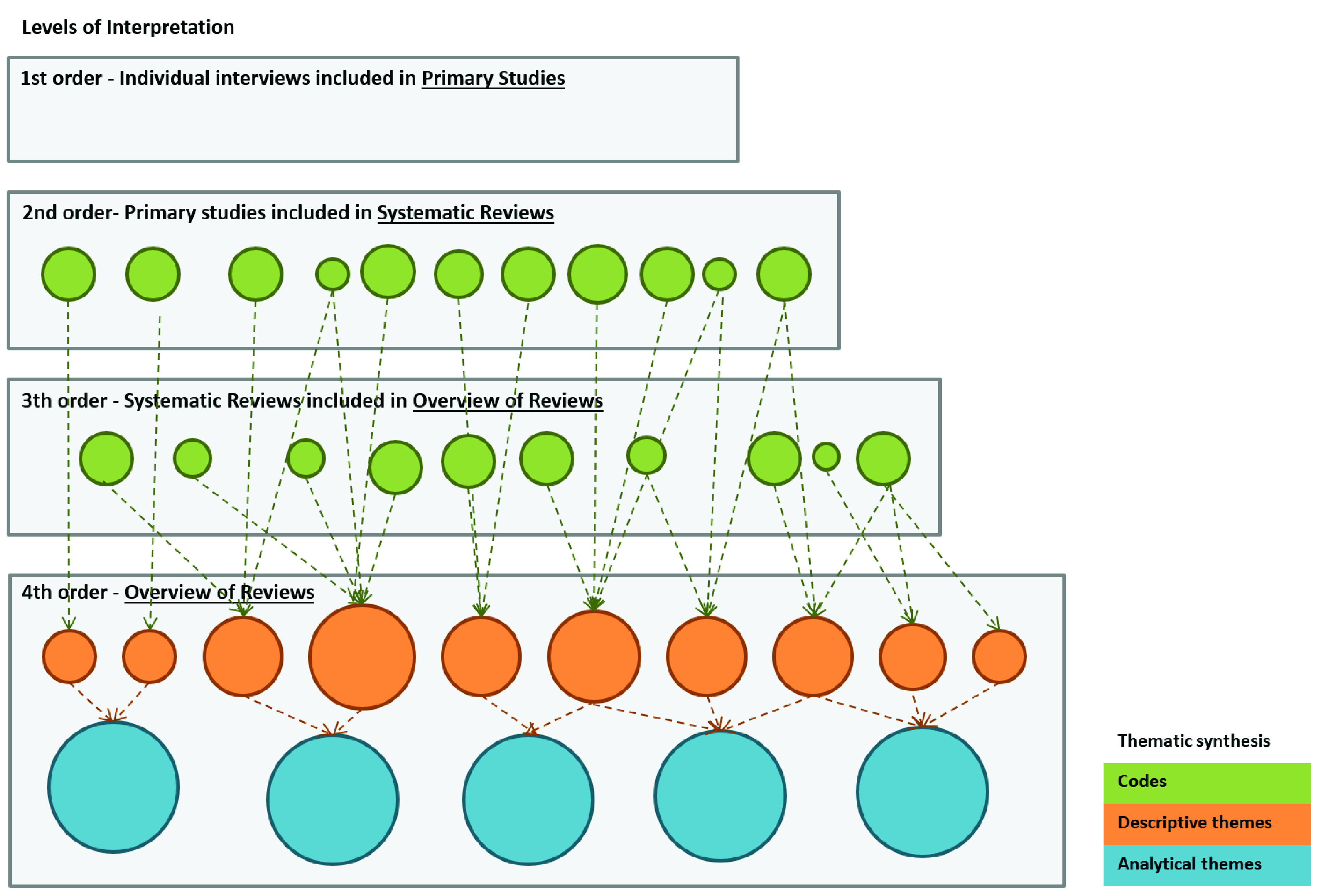
Thematic synthesis stages and levels of interpretation. Levels of interpretation: adapted from Pierce *et al.* ^24^, thematic synthesis stages based on Thomas and Newman ^53^.

We will extract data using Nvivo 12 Pro ^44^. We will design and pilot-test one form for each type of SR (quantitative, qualitative or mixed methods). After initial calibration between pairs of authors, one author will extract data from the included reviews, and a second author will check the data against the full text for accuracy. We will solve disagreements by consensus or with the help of a third author. We will collect the following information from each type of SR:

- General characteristics: country of contacting author, year of publication, aim or phenomenon of interest, search databases, search timeframe, the number of included studies, and the number of participants.
- Methodological characteristics: the type of SR, the method of synthesis, methods of primary studies, the quality assessment tool, the certainty of the evidence assessment tool, and their findings.
- Settings: location/s (countries) of primary studies, and the context of included studies.
- Participants’ main characteristics: type of respondent, the severity of the disease, demographic characteristics, and comorbidities.
- Intervention characteristics: target population/s, support technique/s, support delivery method/s, and expected patient (or caregiver) self-management behaviours.
- Themes or findings related to outcomes of SM interventions: 1) quantitative data, including health utility measures (mean, standard deviation, confidence interval) and the methods applied to obtain them; and 2) qualitative data extraction, this process overlaps with the first stage of thematic synthesis ( Figure 1).

In the extraction process, we will record the extent of relevant information not reported (e.g. information about age), but we will not take additional steps to get further information from primary studies ^29, 45^. For discrepant data (two or more SRs reporting different data for the same primary study), we will extract data from the SR with the highest methodological quality, and if similar methodological quality, from the review with the most updated search ^45^. For discrepant methodological quality assessment (two or more SRs using different tools or reporting different results for the same primary study), we will report both results.

### Assessment of overlapping primary studies

We will assess the overlapping primary studies (studies appearing in more than one review) using a citation matrix ^46^. We will extract the references of the primary studies answering our research question within all selected reviews, calculate the “corrected covered area” (CCA), and describe the extent of overlap (slight [0 to 5], moderate [6 to 10], high [11 to 15], or very high [over 15]) ^47^. We will include all SRs that meet the eligibility criteria regardless of overlap ^48^. We will conduct this assessment using Excel files.

For duplicated data (two or more SRs reporting the same data from the same primary study or with over 25% of overlap within primary studies), we will include data only once, selecting the one with the most complete and detailed data ^48^.

### Assessment of methodological quality of included systematic reviews

We will use the JBI Critical Appraisal Checklist for Systematic Reviews and Research Syntheses to test the quality of the included reviews ^30^. The JBI Checklist, which comprises 11 items, is a tool to assess the methodological quality of both quantitative SRs and QES ^30^. After initial calibration, one author will independently apply this checklist, and a second author will check results to validate scores. We will solve disagreements through consensus or with the help of a third author. We will adopt a previously published scoring method ^50^ that allocates “one point” to positive responses and “zero points” to negative or unclear responses, and we will calculate the percentage of positive responses for each review (sum of positive responses divided by total items tested). The quality of each SR will be ranked based on the following criteria: 0–33% of criteria met (low quality), 34–66% of criteria met (medium quality), and 67% or more of criteria met (high quality) ^50^. We will not exclude SRs based on their quality scores.

### Data synthesis and reporting

To synthesise findings from selected SRs, we will use a mixed-methods three-step approach, with a convergent parallel analysis of quantitative and qualitative evidence ^51^.

***Step 1: Quantitative evidence.*** To analyse and synthesise quantitative findings (utility and non-utility data), we will use narrative synthesis ^52^. We will calculate descriptive statistics (mean, SD for variables with a normal distribution and the median, interquartile range, for other distributions).

For reporting, we will present results in summarised tables (grouping by relevant categories of outcomes). We will plot reported results, e.g. mean values, 95% confidence intervals and I ^2^ statistic for heterogeneity (if pooled), or the range of mean values or other measures (if not pooled) across SRs, the number of studies and the number of respondents for each outcome. Where possible we will present results by country of study, location, type of respondent (patient or caregiver), respondents’ characteristics (sample size, the severity of disease), and methods used. We will perform statistical analyses using STATA software V.14 ^53^.

***Step 2: Qualitative evidence.*** We will analyse and synthesise qualitative data using a thematic synthesis ^49^ approach using NVivo 12 Pro ^44^. The levels of analysis will comprise second-order (primary studies) and third-order SRs constructs ^24, 54^ ( Figure 1). We will not specify any *a priori* theme.

The synthesis process will follow the three stages of thematic synthesis ^49^, the first two taking place concurrently: 1) text coding - line-by-line coding of the text of each SR; 2) developing descriptive themes; 3) re-interpretation and synthesis of this newly organised information, to produce analytical themes that go beyond the findings of the SRs authors (overview of SRs) ( Figure 1). For this step, we will conduct a collaborative analysis with at least three review authors to consider alternative interpretations and ensure that fourth-order constructs remain grounded in the primary studies.

***Step 3: Integration of qualitative and quantitative evidence.*** We will integrate the results of these two sources of evidence in the interpretation phase of results ^55^. For findings informing outcomes, we will organise them according to the COMPAR-EU taxonomy (or to additional outcomes, if applicable) ^39^. For emerging themes informing about feasibility and acceptability of SM interventions, we will develop a framework, according to the classification of analytical themes. The goal of the integration phase is to categorise the importance of outcomes and provide a framework to identify the factors that influence acceptability and feasibility of SM interventions, always from the perspectives of patients and their caregivers. We will present findings using tabular formats, which will inform the development of decision-making tools for the interactive platform of the COMPAR-EU project.

***Sub-group analysis.*** For quantitative evidence and depending on the sufficiency of SRs, we plan to analyse the primary outcomes according to the patient population (e.g. age, the severity of the disease, type of respondent), intervention (i.e. type of SM intervention) and setting (i.e. country). For qualitative evidence, it is not possible to specify the groups in advance, due to the nature of this synthesis.

***Sensitivity analysis.*** If applicable, we will conduct a sensitivity analysis exploring the impact of the quality of the included SRs (low, medium and high) in the final synthesis of findings.

### Patient’s and caregiver’s involvement

After the integration of quantitative and qualitative findings, we will conduct an online survey with patients’ and caregivers’ representatives from each chronic condition. We will ask their feedback on the draft of summaries of perspectives of patients and their caregivers derived from the SRs. We will design a tabular format for the seven subdomains of outcomes with close and open-ended questions assessing: 1) to what extent the overview findings resound with their preferences and experiences; and 2) the relevance of findings for healthcare decision-making on SM interventions from their point of view.

For the recruitment of participants, we will invite patients’ and caregivers’ representatives through the patient’s networks collaborating in the COMPAR-EU project. We aim to include at least five patients with different profiles of gender, age group, and severity of disease and one informal caregiver for each condition. After giving their informed consent, patients will receive the link to complete the survey. Their responses will be analysed based on frequencies, and thematic analysis for the open-ended questions. After discussion within the review working group, we will include their perspectives in the discussion section of each chronic condition manuscript.

### Ethics and dissemination

Ethical approval was obtained by the Clinical Research Ethics Committee of the Avedis Donabedian Research Institute, the coordinator partner of the COMPAR-EU Project (EU 754936), the University Institute for Primary Care Research (IDIAP Jordi Gol) and was signed on March 2018. We will disseminate our findings as conference abstracts, peer-review manuscripts, and in social community media. Patients’ and caregivers’ representatives participating in the draft summaries assessment will receive feedback based on their responses. Our results will inform the development of decision-making tools for the COMPAR-EU Project.

### Study status

Since we planned to conduct the overviews of reviews for the four conditions consecutively, we are in different points on each one: 1) we conducted the searches in the selected databases and selected the initial list of included reviews for the four conditions on March 2019; 2) we finalised the extraction of the quantitative data of SRs reporting health utilities for the four conditions on September 2019; 3) we started the extraction of qualitative data, and thematic synthesis for reviews of T2DM on November 2019 and this process is still in progress; 4) in the upcoming months we will follow with obesity, HF and COPD. Finally, we plan to publish results also in a consecutive manner during the second semester of 2020 and the first of 2021.

## Discussion

Our study will collect the perspectives of patients and their caregivers on SM interventions for four high priority chronic conditions. We will synthesise and integrate quantitative and qualitative findings describing how patients and their caregivers value the importance of outcomes of SM interventions, and the factors that might affect acceptability and feasibility of conducting these SM interventions, based on their preferences and experiences about SM, through the disease trajectory and with SM interventions.

To our knowledge, these will be the first overview of the perspectives of patients and their caregivers on SM interventions for any of these four chronic conditions using a mixed-methods approach ^26^. We will follow the most updated methodological guidance for overviews ^27– 34^.

Each overview will provide new knowledge in a summarised format about the perspectives of patients living with the four selected chronic conditions and their caregivers on SM interventions. This evidence will be readily available to inform decision-making tools (i.e. EtD frameworks and decisions aids) where, in addition to effectiveness and cost-effectiveness findings, the COMPAR-EU project will develop recommendations for SM interventions. More precisely, we will inform about the importance of outcomes of SM interventions, acceptability and feasibility considerations, through quantitative and qualitative data.

Although patients vary greatly in their preferences ^10^, this approach will help to identify subgroups of patients (and caregivers) that might find an acceptable risk-benefit balance for SM interventions. We will also inform about acceptability and feasibility considerations for implementation concerns of these interventions. Our findings will be incorporated into the COMPAR-EU Project final product: an interactive platform that will be designed and adapted to different profiles of end-users, including patients, caregivers, healthcare providers, policymakers, researchers and industry representatives.

Some limitations include that our findings will represent a sample of primary studies published in English already included in SRs. Because of time constraints, we do not plan to update them ^32^, so we may miss the most recent studies. Furthermore, we are not using a specific tool to assess the quality of reporting. Instead, we will describe this aspect as an overall assessment ^32^. Finally, the overall assessment of the certainty of the evidence is out of our scope, since there is still scarce guidance on how to assess this aspect within an overview ^32^.

Our findings will help to characterise better the perspective of patients with chronic conditions and their caregivers on SM interventions, identifying challenges patients face in their daily life, which may inform and help healthcare providers to get a deeper understanding the patients’ experiences and what is important for them. In this way, they will be able to provide support to patients that better fits their needs. For policy and decision-makers, being aware of the perspective of patients will help to design patient-centred strategies to implement SM interventions.

We expect our findings will provide valuable insights to be considered in the design and conduct of future studies of SM interventions for patients living with any of these or similar chronic conditions. These overviews will help to identify gaps in research in this field and areas of potentially redundant research, reducing waste and streamlining the use of limited resources.

## Data availability

### Underlying data

No underlying data are associated with this article.

### Extended data

OSF: The perspectives of patients and their caregivers on self-management interventions for chronic conditions. https://doi.org/10.17605/OSF.IO/GFSA5 ^56^

### Reporting guidelines

OSF: The perspectives of patients and their caregivers on self-management interventions for chronic conditions. https://doi.org/10.17605/OSF.IO/GFSA5 ^56^.

Data are available under the terms of the Creative Commons Attribution 4.0 International license (CC-BY 4.0).
