# Supplementary material for: The perspectives of patients and their caregivers on self-management interventions for chronic conditions: a protocol for a mixed-methods overview
Source: F1000Res. 2021 Nov 29;9:120. Originally published 2020 Feb 18. [Version 2] doi: 10.12688/f1000research.22125.2 (PMC8825946; doi:10.12688/f1000research.22125.2)
Supplement: Peer review history

# Peer review: Reviewer response for version 2

**DOI:** 10.5256/f1000research.79074.r101464

**Published:** 2021-12-01

**Authors:** Edward Zimbudzi

**Affiliations:** 1 Monash Centre for Health Research and Implementation, Monash University, Melbourne, Australia

The authors have addressed my comments.

Is the study design appropriate for the research question?

Yes

Is the rationale for, and objectives of, the study clearly described?

Yes

Are sufficient details of the methods provided to allow replication by others?

Yes

Are the datasets clearly presented in a useable and accessible format?

Not applicable

Reviewer Expertise:

Chronic kidney disease; self-management; patient activation; health related quality of life; dialysis

I confirm that I have read this submission and believe that I have an appropriate level of expertise to confirm that it is of an acceptable scientific standard.

# Peer review: Reviewer response for version 1

**DOI:** 10.5256/f1000research.24400.r84691

**Published:** 2021-06-02

**Authors:** Edward Zimbudzi

**Affiliations:** 1 Monash Centre for Health Research and Implementation, Monash University, Melbourne, Australia

In this protocol, the authors seek to identify, appraise and synthesise evidence regarding patients' and caregivers' perspectives on self-management interventions. To do this, the authors will conduct four mixed-methods overviews specific to patients with chronic obstructive pulmonary disease (COPD), heart failure (HF), obesity and type 2 diabetes mellitus. Appropriate databases will be searched for systematic reviews that meet preidentified criteria followed by well described data extraction and analysis process. 

 Here are my comments/remarks that require clarification;

- The authors may need to make it clear from the onset that they will include patients with only one of the four conditions (if that is the case). It is important to clarify this because a number of patients have more than one condition.
- In line with the point above, patients may also have a chronic condition of interest plus other chronic diseases which are not part of this study. Are they going to be excluded?
- The stage of the disease does affect how patients self-manage as well as outcomes. Authors may need to provide a rationale regarding why they are not considering the stage of the disease as a variable. 

Is the study design appropriate for the research question?

Yes

Is the rationale for, and objectives of, the study clearly described?

Yes

Are sufficient details of the methods provided to allow replication by others?

Yes

Are the datasets clearly presented in a useable and accessible format?

Not applicable

Reviewer Expertise:

Chronic kidney disease; self-management; patient activation; health related quality of life; dialysis

I confirm that I have read this submission and believe that I have an appropriate level of expertise to confirm that it is of an acceptable scientific standard, however I have significant reservations, as outlined above.

# Response

**Published:** 2021-11-02

**Authors:** Ena Niño de Guzman

**Affiliations:** Iberoamerican Cochrane Center, Spain

**Eligibility criteria**

 We will include SRs that included studies in patients with chronic conditions of interest (COPD, HF, obesity and T2DM) and without restricting by 1) disease severity, 2) the number of conditions of interest (e.g., > 1 of the chronic conditions of interest), or 3) the number of chronic conditions (e.g., *≥* 1 of the chronic conditions of interest plus *≥* 1 of other chronic conditions, except cancer or severe mental disorders). We will collect and analyse data considering these criteria if data is available.

 We will collect and analyse data considering disease severity and the number and types of chronic conditions if data is available. If SRs included more than one of the chronic conditions of interest, we will collect and analyse data of each condition in the corresponding overview.

 We have modified the text in the "Methods and analysis - Eligibility criteria - Population/perspectives" section to clarify the reviewer's concern. The text reads: "We will limit inclusion to SRs of studies conducted in adult patients (18 or older) with one or more of the chronic conditions of interest (COPD, HF, obesity and T2DM), without restricting by the severity of the disease. We will consider SRs assessing multiple chronic conditions (e.g., patients with more than one of the chronic conditions of interest or patients with chronic conditions of interest plus other chronic conditions). We will exclude SRs focused on patients with the chronic conditions of interest and cancer comorbidity or severe mental disorders."

 We have modified the text in the "Methods and analysis - Data synthesis and reporting - Sub-group analysis" section to clarify the reviewer's concern. The text reads: "For quantitative evidence and depending on the sufficiency of SRs, we plan to analyse the primary outcomes according to the patient population (e.g., age, the severity of the disease, number and types of chronic conditions, and type of respondent), intervention (e.g., type of SM intervention) and setting (e.g., country)".

# Peer review: Reviewer response for version 1

**DOI:** 10.5256/f1000research.24400.r66199

**Published:** 2020-08-24

**Authors:** Milena Vainieri

**Affiliations:** 1 Management and Health Laboratory, Sant'Anna School of Advanced Studies of Pisa, Pisa, Italy

The article presents the study protocol for a systematic review of the reviews dealing with 4 main chronic diseases.

 The methods that this group of authors intend to apply are innovative and comprehensive.

 The only minor suggestions regard the language proofreading (for instance see the use of connects as a noun) and if the authors expect to publish a review per disease or a unique comprehensive review also comparing the 4 (potentially overlapping) diseases.

Is the study design appropriate for the research question?

Yes

Is the rationale for, and objectives of, the study clearly described?

Yes

Are sufficient details of the methods provided to allow replication by others?

Yes

Are the datasets clearly presented in a useable and accessible format?

Not applicable

Reviewer Expertise:

Health service research, healthcare management

I confirm that I have read this submission and believe that I have an appropriate level of expertise to confirm that it is of an acceptable scientific standard.

# Response

**Published:** 2021-11-02

**Authors:** Ena Niño de Guzman

**Affiliations:** Iberoamerican Cochrane Center, Spain

Comment 1: Language proofreading and publication plan

**Response 1**

 We have reviewed English grammar correctness and made some minor edits. We plan to publish one overview per disease. We have modified the text in the " Study status" section to clarify the reviewer's concern. The text reads: “... Finally, we plan to publish results also in a consecutive manner, one manuscript per condition, during the second semester of 2021 and the first of 2022.”
